# Progressively enlarging eyelid nodule

**DOI:** 10.1016/j.jdcr.2024.06.006

**Published:** 2024-06-18

**Authors:** Rachel Chang, Kim Tran, Christof Erickson, Zhe Hou

**Affiliations:** a Rush University Medical Center, Chicago, Illinois; b Imperial Dermatology, Brea, California; c Compass Dermatopathology, San Diego, California

**Keywords:** nodular fasciitis, recurrence, subcutaneous nodule, trauma

## History

**Fig 1:**
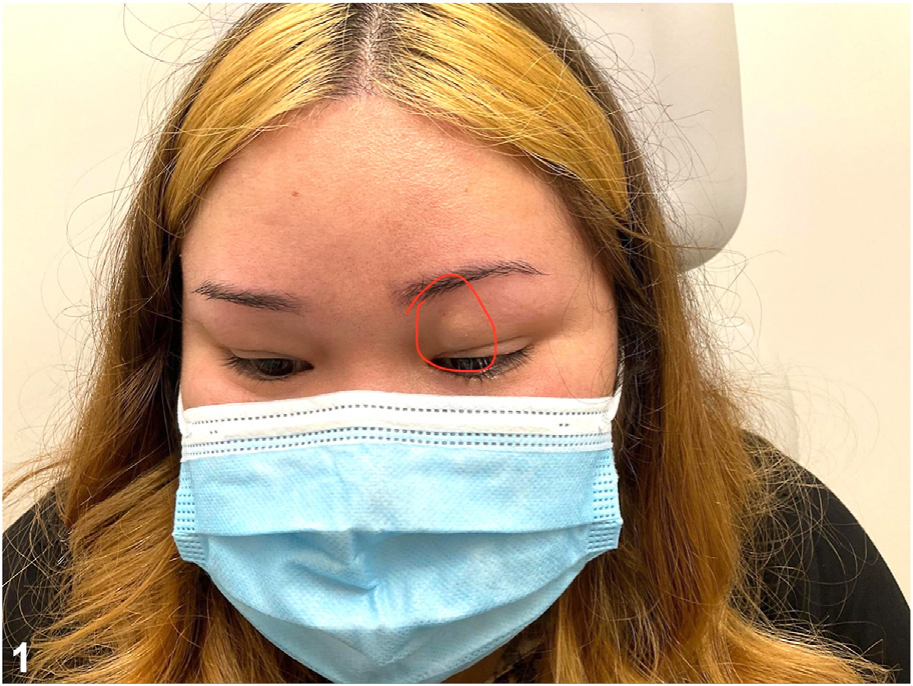

**Fig 2:**
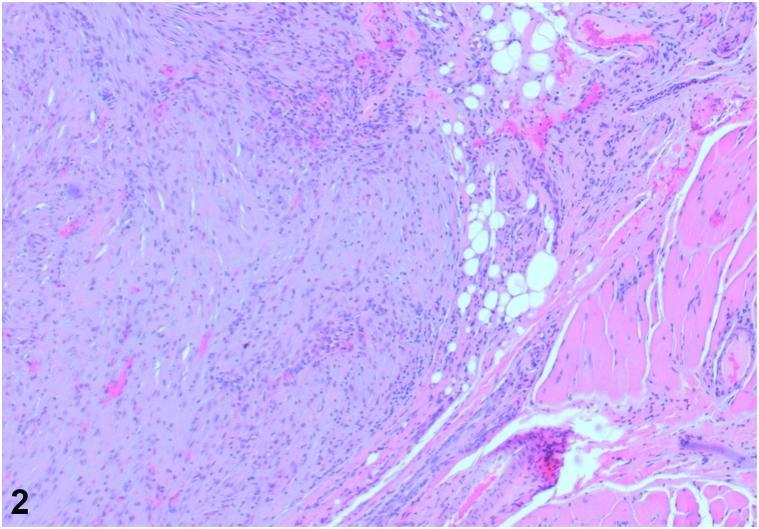

**Fig 3:**
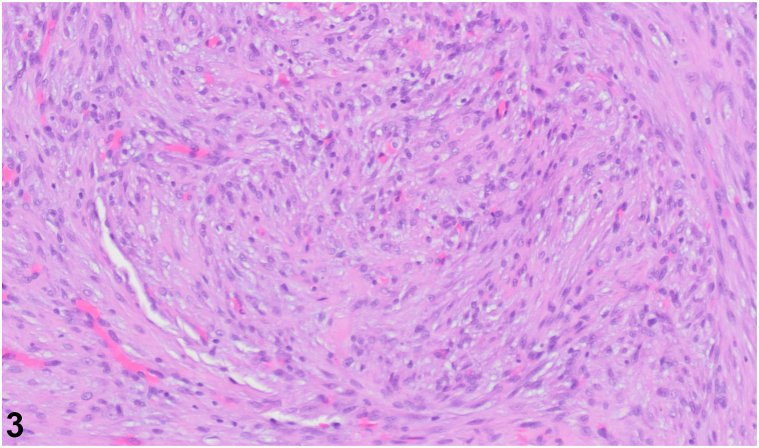

A 29-year-old female presented with a 2-month history of an enlarging, tender mass in the left eyelid after trauma to the periorbital region (Fig 1). The lesion resulted in mechanical ptosis of the eye, with associated vision obstruction. Physical examination revealed a 1.5 cm soft, nonmobile nodule. Excisional biopsy was performed. Microscopy revealed an irregular proliferation of spindled to ovoid cells with fibromyxoid stroma and scattered thick collagen fibers in the subcutis and skeletal muscle (Fig 2). A myxoid stroma with extravasated red blood cells is also observed (Fig 3). Immunohistochemistry stained positive for SMA, NKI-C3, Caldesmon, CD68, and Desmin.

**Question 1: What is the most likely diagnosis?**

A. Dermatofibroma
B. Nodular fasciitis
C. Primary cutaneous leiomyosarcoma
D. Foreign body granuloma
E. Cutaneous meningioma

**Answers:**

A. Dermatofibroma – Incorrect. Dermatofibroma typically presents with spindle- or round-shaped fibroblasts and histiocytes, hyalinized collagen bundles, chronic inflammatory cells, macrophages, and multinucleated Touton giant cells.^1^ Dermatofibromas are longstanding, without history of rapid growth. The overlying skin characteristically exhibits the “dimple sign” upon lateral compression.
B. Nodular fasciitis – Correct. Nodular fasciitis is a benign proliferation of fibroblasts and myofibroblasts typically presenting as a rapidly-growing lesion on the trunk and upper extremities.^2^ The t(17;22) translocation and subsequent binding of the USP6 coding region to the MYH9 promoter region increases USP6 expression, driving the tumorigenesis of nodular fasciitis. Histologically, pleomorphic-arranged spindle cells in a myxoid stroma are typically observed.
C. Primary cutaneous leiomyosarcoma – Incorrect. Primary cutaneous leiomyosarcoma presents with pleomorphic spindle cells, nuclear atypia, and high mitotic activity. Nodular fasciitis typically does not exhibit nuclear atypia. Nodular fasciitis has a predilection for young and middle-aged adults, while cutaneous leiomyosarcoma commonly presents in adults above 65 years of age.
D. Foreign body granuloma – Incorrect. While trauma can be a risk factor for both foreign body granuloma and nodular fasciitis, foreign body granuloma begins with acute inflammation and erythema at the entry site of a foreign material. Granulomas are characterized by epithelioid macrophages, multinucleated giant cells, and can have central necrosis which is not present in nodular fasciitis.
E. Cutaneous meningioma – Incorrect. Cutaneous meningiomas are slow-growing soft tissue tumors commonly found on the scalp, face, or neck. On histology, epithelioid cells are mixed with individual cells that have elongated or oval-shaped nuclei and open chromatin.

**Question 2: Which of the following statements regarding the presentation of this diagnosis is correct?**

A. Females are more likely to experience this condition than males
B. Older age is a risk factor for developing this condition
C. Lesions are most commonly found on the head and neck
D. Recurrence of lesions following treatment is common
E. Lesions typically present as a single painless mass

**Answers:**

A. Females are more likely to experience this condition than males – Incorrect. The incidence rate of this condition is equal between males and females.^3^
B. Older age is a risk factor for developing this condition – Incorrect. Nodular fasciitis typically affects younger adults, with the majority of cases occurring in individuals between 20 and 40 years of age.
C. Lesions are most commonly found on the head and neck – Incorrect. Nodular fasciitis typically occurs in the trunk or upper extremities. Only approximately 7% of lesions occur in the head and neck area, with less than 1% of all cases presenting in the orbital region.^4^
D. Recurrence of lesions following treatment is common – Incorrect. Recurrence is rarely observed. In an analysis of 250 patients treated for this condition, only one case of recurrence occurred, which was fully resolved after a second treatment.^5^
E. Lesions typically present as a single painless mass – Correct. Nodular fasciitis presents as a single solid, rubbery, firm mass around 2 cm that is usually painless, but can be tender to touch.

**Question 3: What is the definitive treatment option in this case?**

A. Simple excision or intralesional steroids
B. Oral doxycycline
C. Radiation or ablative therapies
D. Electrodessication and curettage
E. Chemotherapy with doxorubicin and ifosfamide

**Answers:**

A. Simple excision or intralesional steroids – Correct. Treatment of nodular fasciitis consists of simple excision, which usually results in permanent resolution.^2^ Intralesional steroids may be used as an alternative method to excision in cases where a complete excision is difficult or used in esthetically important areas such as the head and neck.^4^
B. Oral doxycycline – Incorrect. Nodular fasciitis is not an infectious process, so it would not require antibiotic treatment.
C. Radiation or ablative therapies – Incorrect. Radiotherapy and ablative therapies would not be warranted for nodular fasciitis. Due to its high cellularity, increased mitotic activity, and local infiltration of surrounding tissue with lack of a true capsule, along with mild cellular pleomorphism, nodular fasciitis can be mistaken as a malignant tumor.^4^ Misdiagnosis of nodular fasciitis as malignant has led to delay in management and unnecessarily aggressive treatment in some patients.
D. Electrodessication and curettage – Incorrect. Electrodessication and curettage is not typically used to manage nodular fasciitis.
E. Chemotherapy with doxorubicin and ifosfamide – Incorrect. Combination anthracycline based therapy would not be warranted for nodular fasciitis. It would be appropriate for treatment of soft tissue sarcomas.

## Conflicts of interest

None disclosed.
